# Substrate Stiffness Regulates Cholesterol Efflux in Smooth Muscle Cells

**DOI:** 10.3389/fcell.2021.648715

**Published:** 2021-05-18

**Authors:** Xiuli Mao, Yiling Tan, Huali Wang, Song Li, Yue Zhou

**Affiliations:** ^1^School of Biomedical Engineering, Shanghai Jiao Tong University, Shanghai, China; ^2^Department of Bioengineering and Department of Medicine, University of California, Los Angeles, Los Angeles, CA, United States

**Keywords:** vascular smooth muscle cells, substrate stiffness, cholesterol, foam cells, cholesterol efflux

## Abstract

The infiltration and deposition of cholesterol in the arterial wall play an important role in the initiation and development of atherosclerosis. Smooth muscle cells (SMCs) are the major cell type in the intima. Upon exposure to cholesterol, SMCs may undergo a phenotype switching into foam cells. Meanwhile, the pathological processes of the blood vessel such as cholesterol deposition and calcification induce the changes in the substrate stiffness around SMCs. However, whether substrate stiffness affects the cholesterol accumulation in SMCs and the formation of foam cells is not well-understood. In this study, SMCs were cultured on the substrates with different stiffnesses ranging from 1 to 100 kPa and treated with cholesterol. We found that cholesterol accumulation in SMCs was higher on 1 and 100 kPa substrates than that on intermediate stiffness at 40 kPa; consistently, total cholesterol (TC) content on 1 and 100 kPa substrates was also higher. As a result, the accumulation of cholesterol increased the expression of macrophage marker CD68 and downregulated SMC contractile marker smooth muscle α-actin (ACTA2). Furthermore, the mRNA and protein expression level of cholesterol efflux gene ATP-binding cassette transporter A1 (ABCA1) was much higher on 40 kPa substrate. With the treatment of a liver X receptor (LXR) agonist GW3965, the expression of ABCA1 increased and cholesterol loading decreased, showing an additive effect with substrate stiffness. In contrast, inhibition of LXR decreased ABCA1 gene expression and increased cholesterol accumulation in SMCs. Consistently, when ABCA1 gene was knockdown, the cholesterol accumulation was increased in SMCs on all substrates with different stiffness. These results revealed that substrate stiffness played an important role on SMCs cholesterol accumulation by regulating the ABCA1 expression. Our findings on the effects of substrate stiffness on cholesterol efflux unravel a new mechanism of biophysical regulation of cholesterol metabolism and SMC phenotype, and provide a rational basis for the development of novel therapies.

## 1. Introduction

Atherosclerosis is a multi-factorial process involving significant changes in microenvironment and vascular cell phenotypes (Falk, 2006; Libby et al., 2011). The disorder of lipid metabolism leads to the development of atherosclerotic plaque, which forms a necrotic core containing cells with lipid (foam cells). It has been generally accepted that most foam cells were originated from macrophages, which derives from the circulating monocytes (Yu et al., 2013). However, vascular smooth muscle cells (SMCs) have also been shown to participate in foam cell formation (Allahverdian et al., 2012). The uptake and accumulation of excess cholesterol in SMCs is an external driver to convert SMC phenotype. SMCs lose their contractile makers, such as smooth muscle α-actin (ACTA2), and increase the expression of markers of macrophage phenotype such as CD68 (Gomez and Owens, 2012; Chaabane et al., 2014). This conversion plays a critical role in the composition and stability of the plaque and contributes to the development and progress of atherosclerosis.

During the formation of atherosclerotic plaque, with the accumulation of cholesterol and calcification, the stiffness of the blood vessels changes. The stiffness of normal aorta in rabbit was about 40 kPa, while the local elastic modulus of atherosclerotic lesions of rabbit thoracic aortas decreased at the early stage and increased at later stage with the development of atherosclerosis (Matsumoto et al., 2002). In apolipoprotein E-deficient mice, the stiffness of lipid rich areas in the blood vessel was 5.5 ± 3.5 kPa while the stiffness of the fibrous caps was almost 59.4 ± 47.4 kPa, even rising to 250 kPa (Tracqui et al., 2011). The stiffness of aorta in monkeys was around 20–40 kPa (Sehgel et al., 2015). These findings suggest that the physiological stiffness of aorta is about 40 kPa and the pathological changes may decrease the stiffness to a level as low as 1 kPa and increase the stiffness to 100 kPa or higher. Meanwhile, vascular endothelial cells and SMCs can sense and respond to such stiffness changes in the vascular wall, causing phenotypic switching of these cells (Rudijanto, 2007; Tian et al., 2019). It has been reported that membrane cholesterol and substrate stiffness can synergistically affect the biomechanical properties and the remodeling of the cytoskeleton of SMCs. Atomic force microscopy revealed that substrate stiffness also regulated the stiffness of SMCs themselves, as well as their adhesion behavior mediated by α5β1-integrin (Sanyour et al., 2019). However, it is not known whether the stiffness change in the microenvironment affects the cholesterol metabolism in SMCs and their phenotype change.

Cholesterol is an essential component of cell membrane to maintain the membrane integrity, fluidity, and metabolic function of the cells (Maxfield and Tabas, 2005; Gelissen and Brown, 2017). Cellular cholesterol homeostasis is controlled by the precise regulation of the uptake, synthesis, and efflux of cholesterol, where cholesterol efflux is a major part of reverse cholesterol transport (RCT) (Litvinov et al., 2016). RCT is the only way to remove excess cholesterol from the cells in peripheral tissues and transport it to liver and intestine, which can prevent cholesterol accumulation in vascular cells and inhibit the development of atherosclerosis (Simons and Ikonen, 2000; van der Wulp et al., 2013). The membrane cholesterol transporters, such as ATP-binding cassette transporter A1 (ABCA1), participates in RCT to transport the excessive phospholipids and cholesterol to the outside of the cells (Oram and Heinecke, 2005; Liao and McLachlan, 2018). On a high-cholesterol diet, ABCA1-deficient mice showed more cholesterol accumulation and more foam cells formation than wild-type mice (Choi et al., 2009). The sterol-responsive nuclear receptor, liver X receptor (LXR), is a vital transcription factor being activated in response to excess cellular cholesterol (Im and Osborne, 2011; Rasheed and Cummins, 2018). It has been reported that LXR agonists can facilitate RCT and protect mice against atherosclerosis (Joseph et al., 2002). Since the focus of cellular cholesterol homeostasis has been carried out in the monocyte-derived macrophages, little has been explored from the aspect of the cholesterol homeostasis in SMCs. There is evidence that the formation of SMC-derived foam cells in the human coronary arteries is partially due to their reduced ability to remove excess cholesterol through ABCA1 (Allahverdian et al., 2014). However, it is not clear whether substrate stiffness could regulate SMCs cholesterol accumulation and efflux in the pathogenesis of atherosclerosis.

To address this question, we varied the stiffness of extracellular substrate ranging between 1 and 100 kPa, and investigated the effects of substrate stiffness on cholesterol accumulation and efflux in SMCs and their interactions with LXR signaling pathway, demonstrating the crosstalk between biophysical and biochemical signaling in the regulation of SMC functions.

## 2. Materials and Methods

### 2.1. Preparation of Substrates With Different Stiffness

Substrates with different stiffness were prepared as described previously by adjusting the ratio of 40% acrylamide to 2% bis-acrylamide (w/v) (Sangon Biotech, China) (Chen et al., 2019; Tian et al., 2019). Tetramethylethylenediamine (TEMED, Klamar, China) and 10% ammonium persulfate solution (APS, Sinopharm, China) were used as cross-linking reagent. Before polymerization, 200 μl mixture was added onto a glass slide pre-treated with gel slick (Lonza, Switzerland), and a piece of silicified cover slip was put upon the gel solution to make a sandwich. After 5 min, the substrates were treated with sulfo-SANPAH (Thermo Fisher, USA) and coated with 1 mg/mL collagen I (Corning, USA) to facilitate the cell adhesion. Finally, the substrate was sterilized by 75% alcohol and UV before cell culture.

### 2.2. Cell Culture

Human aortic smooth muscle cells (SMCs) were purchased from ScienCell Research Laboratories (ScienCell, USA) and used for experiments at passages 5–10. Cells were cultured in SMC medium (ScienCell, USA) supplemented with 2% fetal bovine serum, 1% penicillin/streptomycin, and 1% smooth muscle cell growth supplement (SMCGS) in a 37°C incubator (Corning, USA) with 5% *CO*_2_.

To induce the foam cell state, SMCs were primed by Chol: MβCD complex (20 μg/ml) (Sigma, USA), a “water-soluble cholesterol” for 72 h.

In the inhibition experiments, SMCs were treated with GSK2033 (MedChemExpress, China), a liver X receptor (LXR) inhibitor, at a final concentration of 1 μM in the culture medium for 72 h. DMSO was used as control.

In the agonist experiments, SMCs were treated with GW3965 (Sigma, USA), a selective agonist of LXR, at a final concentration of 1 μM in the culture medium for 72 h. DMSO was used as control.

### 2.3. Cell Viability Test

To optimize the concentration of Chol: MβCD, SMCs were seeded in a 96-well plate and treated with a concentration gradient of Chol: MβCD (0, 10, 20, 40, and 80 μg/ml) for 72 h at standard culture condition. Then, the culture media was removed and the cell were incubated with CCK-8 reagent (DOJINDO, Japan) for 1 h. Optical density (OD) at 450 nm of each well was determined using a plate-reader (Bio Tek, Synergy 2, USA). The OD of the experiment and the control groups were both corrected by subtracting the OD of the blank. Cell viability was expressed as the percentage OD_450 *nm*_ of experimental group to that of the control group.

### 2.4. Cellular Cholesterol Uptake and Quantitation

Oil Red O staining was used to quantify the amount of cholesterol in the SMCs. Briefly, after cholesterol loading, SMCs were fixed in 4% (w/v) paraformaldehyde (PFA) and incubated in 60% isopropanol for 3 min. Then, cells were stained with Oil Red O and Harris' hematoxylin. Images were captured by a light-microscope (DMI3000B, Leica, Germany). The cellular cholesterol was extract by isopropanol and OD value at 520 nm was determined to represent the cholesterol concentration.

The cholesterol uptake by SMCs was identified by BODIPY 493/503 staining (Qiu and Simon, 2016). After 20 μg/ml cholesterol treatment for 72 h, SMCs were washed 3 times with Dulbecco's phosphate buffered saline (DPBS) and 2 μM BODIPY 493/503 was added (Cayman, USA) for 15 min at 37°C in dark. Then, cells were fixed in 4% (w/v) PFA for 15 min, and incubated with DAPI for 5 min to stain the nucleus. Images were captured by a laser scanning confocal microscope (Leica, TCS SP5 II, Germany). Cholesterol uptake was quantified by relative fluorescence intensity (FI).

### 2.5. Cellular Cholesterol and Protein Determination

Cellular total cholesterol (TC) was determined by TC assay kit (NanJing Jiancheng Bioengineering Institute, China) according to manufacturer's instructions. The cellular cholesterol concentration was normalized by total protein content determined by BCA Protein Assay Kit (Thermo Scientific, USA).

### 2.6. Cholesterol Efflux Determination

Cholesterol concentration is proportional to the intensity of the 20S-methyl-21-[(7-nitro-2,1,3β-benzoxadiazol-4-yl) amino]-pregn-5-en-3-ol (NBD cholesterol) (J&K Scientific, China) fluorescence signal. After cholesterol loading, the SMCs were incubated with NBD cholesterol for 8 h and Apoa1 for additional 8 h to induce cholesterol efflux. The culture medium comprised of the cholesterol efflux was collected and the SMCs were lysed with 2% Triton X-100 to release the cholesterol remained in the cells. Both the efflux and the supernatant from the lysed cells (non-efflux) were subject for fluorescence intensity (FI) measuring at 528±20 nm (with 485±20 nm excitation wavelength). Cholesterol efflux rate = efflux FI / (efflux FI + non-efflux FI) × 100%.

### 2.7. Real-Time Quantitative Polymerase Chain Reaction (RT-qPCR)

Total RNA was extracted with the RNA simple Total RNA Extraction kit (TIANGEN, China) and reverse transcribed into cDNA by using the Fastking RT kit (with gDNase) (TIANGEN, China), following the manufacturer's instruction. The mRNA level was determined by RT-qPCR (Thermo Fisher Scientific, USA) using the Talent qPCR PreMix Kit (SYBR Green) (TIANGEN, China) and the specific primer pairs was listed in Table 1. All data were normalized to GAPDH and expressed as fold change over the controls.

**Table 1:** RT-qPCR primer sequences.

**Primer name**	**Forward primer (5'-3')**	**Reverse primer (5'-3')**
GAPDH	GGGAAGGTGAAGGTCGGAGT	GGGGTCATTGATGGCAACA
ABCA1	CAGGAGGTGATGTTTCTGACC	CGCAGACAATACGAGACACAG
ACTA2	GGACATCAAGGAGAAACTGTG	CCATCAGGCAACTCGTAACT
CD68	TGCTTCTCTCATTCCCCTATG	GGTAGACAACCTTCTGCTGGA
LGALS3	CATGCTGATAACAATTCTGGG	GGTTAAAGTGGAAGGCAACAT

### 2.8. Western Blotting

Total protein of SMCs was extracted by the plus RIPA lysis buffer (Beyotime Biotechnology, China) with protease inhibitor. BCA Protein Assay Kit (Thermo Scientific, USA) was used to quantify the protein concentration. Then, equal amount of the total cell lysates was loaded for SDS-PAGE electrophoresis, and transferred to PVDF membrane. After 10% BSA blocking, the membrane was incubated with primary antibody and incubated overnight at 4°C. The primary antibodies used in this research are ACTA2 (Abcam, UK), CD68 (Abcam, UK), ABCA1 (Novus, USA), α-Tubulin (Cell Signaling Technology, USA), and GAPDH (Cell Signaling Technology, USA). After removing the primary antibody, HRP-conjugated secondary antibodies (Cell Signaling Technology, USA) were added and incubated with the membrane for 1 h at room temperature and visualized by Immobilon western chemiluminescent HRP substrate (Merck Millipore, Germany). The protein expression was normalized by the expression of α-Tubulin or GAPDH in respective samples.

### 2.9. Immunofluorescence Staining

To characterize the SMCs phenotype, cells were fixed in 4% (w/v) PFA, and incubated with primary antibodies of ACTA2 or CD68 overnight at 4°C. Then, cells were washed by DPBS and incubated with the fluorescent labeled secondary antibodies accordingly for 1 h at room temperature. Finally, cell nucleus was stained with DAPI for 5 min. Images were captured under a laser scanning confocal microscope (Leica, TCS SP5 II).

### 2.10. siRNA Transfection

SMCs were seeded on substrates with different stiffness overnight to reach 50–70% confluency before transfection. The negative siRNA control (NC) or siRNA targeting ABCA1 (siABCA1) (Guangzhou RiboBio, China) was transfected into SMCs at a concentration of 50 nM in the culture medium according to manufacturer's instructions. Twenty-four hours after the transfection, cholesterol was added into the cell culture medium and the cells were treated by cholesterol for 24 h.

### 2.11. Statistical Analysis

Unless otherwise indicated, the results were shown as mean ± SEM (*n* = 3), and student *t*-test was used for pairwise comparisons for the same stiffness between groups and the different stiffness within groups. For all cases, *p* < 0.05 was considered statistically significant. Data were analyzed by GraphPad Prism 6.0 software.

## 3. Results

### 3.1. Cholesterol Accumulation Induced SMCs Phenotype Switching to a Foam Cell-Like State

**Figure 1:**
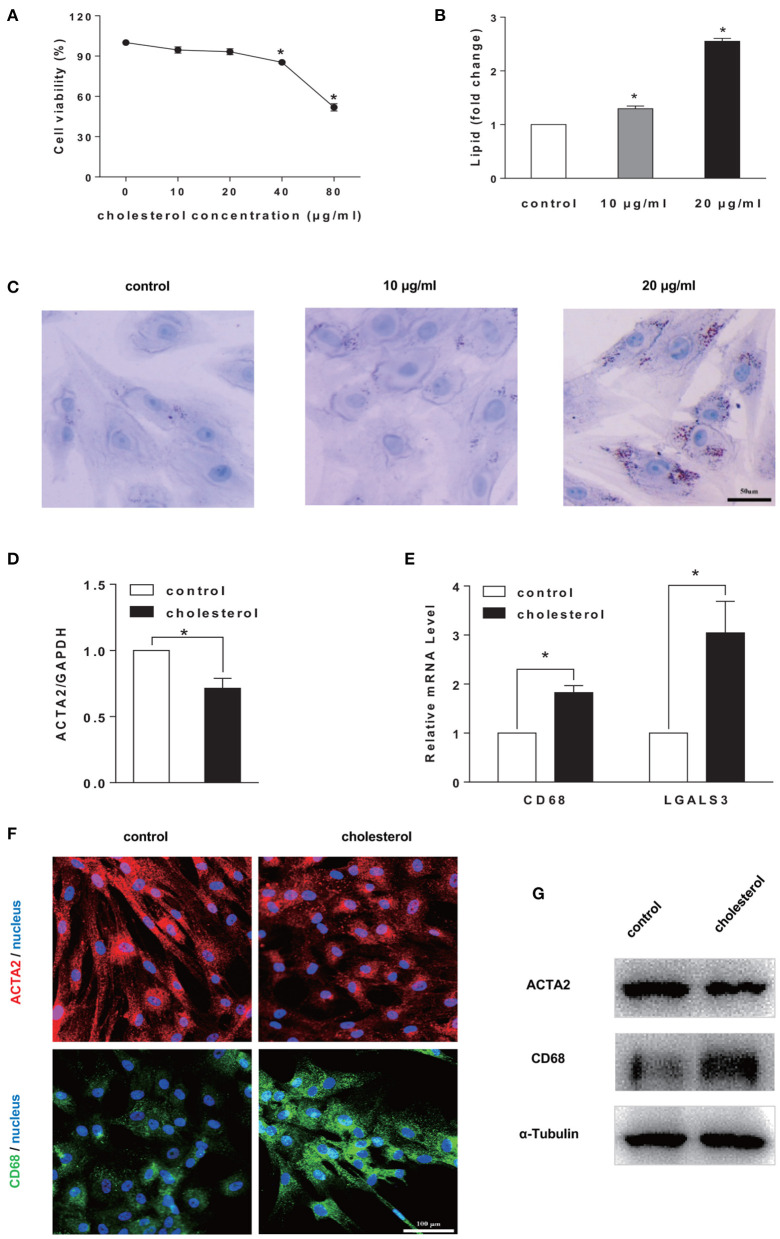
Cholesterol accumulation induced SMCs phenotype switching to a foam cell-like state. **(A)** Cell viability test. SMCs were treated by Chol: MβCD at different concentrations for 72 h. CCK-8 was used to determine cell viability (*n* = 3). **(B,C)** Cellular cholesterol amount determination. Oil Red O staining (in **C**) (scale bar = 50 μm) and quantitation by colorimetric analysis (*n* = 3) (in **B**). **(D,E)** SMCs were treated with 20 μg/ml Chol: MβCD for 72 h, the mRNA expression level of SMC marker (ACTA2) and macrophage markers (CD68 and LGALS3) was measured by RT-qPCR. GAPDH was used as the internal control for normalization (*n* = 3). Fold change is the ratio of SMCs gene expression with and without (control) cholesterol treatment (*n* = 3). **(F)** ACTA2 (red) and CD68 (green) protein expression was examined by immunofluorescence staining (blue: DAPI staining of nucleus, scale bar = 100 μm). **(G)** ACTA2 and CD68 protein expression was analyzed by Western blotting analysis. The expression of α-Tubulin was used as the internal control (*n* = 3). ^*^p < 0.05.

To confirm the effects of loading on SMCs phenotype switching, Chol: MβCD, a “water-soluble cholesterol” that can be efficiently and quickly uptaken by cells, was used to treat SMCs (Christian et al., 1997; Qin et al., 2006). The cholesterol concentration was titrated to show that little cytotoxicity was detected at 20 μg/ml (or lower) after 72 h (Figure 1A). Meanwhile, the cellular uptake of cholesterol was confirmed by Oil Red O staining and quantitation. After Chol: MβCD treatment for 72 h, 20 μg/ml group showed more cholesterol droplets than the other two groups (Figure 1C). In addition, Oil Red O stained cellular cholesterol was extracted by isopropanol and the OD values were compared (Figure 1B).

Furthermore, to characterize the SMCs phenotype switching from SMCs to foam cell-like state, the gene expression level of SMC maker (ACTA2) and macrophage marker gene (CD68 and LGALS3) were analyzed by RT-qPCR. The continuous cholesterol accumulation significantly reduced ACTA2 expression and promoted CD68 and LGALS3 expression (Figures 1D,E). Moreover, ACTA2 and CD68 expression was confirmed by immunostaining and Western blotting analysis (Figures 1F,G).

### 3.2. Substrate Stiffness Regulated Cholesterol Level in SMCs

**Figure 2:**
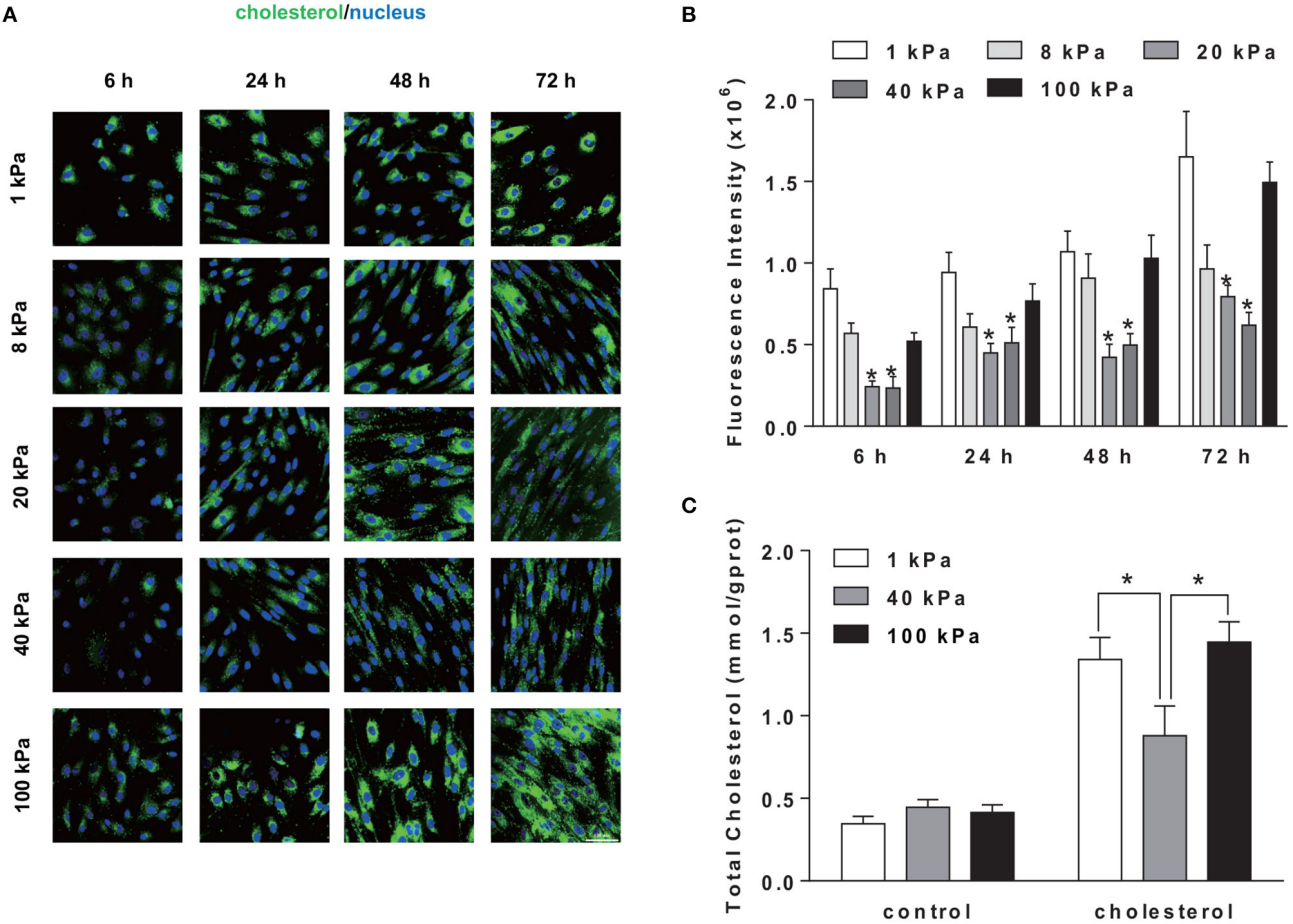
Substrate stiffness regulated cholesterol level in SMCs. SMCs were seeded on substrate of different stiffness (1, 8, 20, 40, 100 kPa) at different time points (6, 24, 48, 72 h). **(A)** The cholesterol level in SMCs (green: BODIPY 493/503 staining of cholesterol; blue: DAPI staining of nucleus, scale bar = 100 μm). **(B)** The level of cholesterol quantified by the fluorescence intensity, which is the total green fluorescence intensity normalized by the number of the cell nucleus (determined by DAPI). ^*^Indicates for comparison with 1 and 100 kPa *p* < 0.05. **(C)** After treated with 20 μg/ml of Chol: MβCD for 72 h, cellular total cholesterol (TC) was quantified by TC Kit and OD_510 *nm*_ was determined by a plate reader (*n* = 3). Cells without treatment were used as the control. ^*^p < 0.05.

To explore the possible influence of substrate stiffness on SMCs cholesterol level, a lipophilic fluorescent probe, BODIPY 493/503, was used to label cellular neutral lipid contents. SMCs were seeded on substrate of different stiffness and treated by cholesterol at indicated times points. The cholesterol accumulation in SMCs showed a time-dependent increase during 72 h and significant difference at different substrate stiffness (Figures 2A,B). At the substrate stiffness of 1 and 100 kPa, cholesterol levels in SMCs were significantly higher than other groups at all time points. This was further confirmed by the quantitation of total cholesterol (TC) level where 1, 40, and 100 kPa were selected as representative stiffnesses (Figure 2C). These results suggested that the stiffness of the substrate affected the accumulation of cholesterol in SMCs.

### 3.3. Substrate Stiffness Affected SMCs Phenotypic Transformation

**Figure 3:**
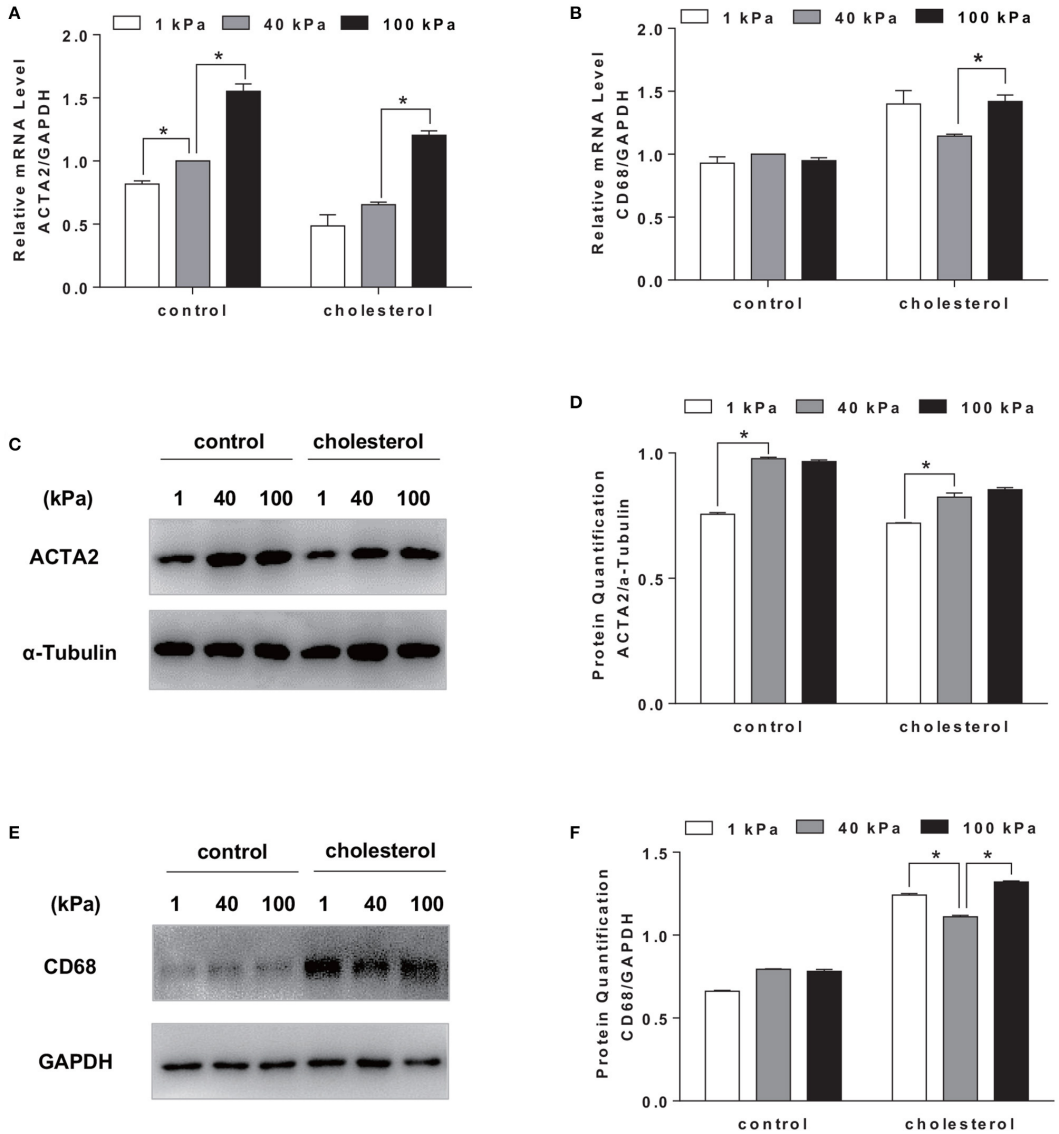
Substrate stiffness affected SMCs phenotypic transformation. SMCs were seeded on substrate with different stiffness and treated with 20 μg/ml Chol: MβCD for 72 h. SMCs without treatment were used as control. **(A,B)** ACTA2 and CD68 gene expression level was measured by RT-qPCR. GAPDH was used as the internal control for normalization (*n* = 3). **(C,D)** ACTA2 protein expression was analyzed by Western blotting analysis. The expression of α-Tubulin was used as the internal control. Quantitation of the protein expression was calculated as the ratio of target protein to α-Tubulin (*n* = 3). **(E,F)** CD68 protein expression was analyzed by Western blotting analysis. The expression of GAPDH was used as the internal control. Quantitation of the protein expression was calculated as the ratio of target protein to GAPDH (*n* = 3). ^*^p < 0.05.

We further determined whether the changes in substrate stiffness would affect the SMCs phenotype. ACTA2 was chosen to represent the contractile phenotype of the SMCs and CD68 as a marker for macrophage phenotype. RT-qPCR results showed that there was a decrease in gene expression of SMC contractile maker ACTA2 and an increase in macrophage marker CD68 (Figures 3A,B) upon cholesterol treatment. While ACTA2 expression increased with substrate stiffness, the suppressive effect of cholesterol was consistent for all stiffness (Figure 3A). The treatment of cholesterol demonstrated a very strong external force to drive SMCs to express stronger macrophage phenotype. However, consistent with the low cholesterol accumulation on 40 kPa substrate, cholesterol treatment demonstrated a significantly lower CD68 expression on 40 kPa compared with 1 and 100 kPa (Figure 3B), implying that substrate stiffness exerted a regulatory effect on SMC phenotype. The protein expression level of both ACTA2 and CD68 was consistent with the mRNA level (Figures 3C,D). SMCs on the 40 kPa substrate showed a relatively smaller change in macrophage marker expression (Figures 3E,F), suggesting less phenotypic change occurred on 40 kPa substrate.

### 3.4. Substrate Stiffness Affected Cholesterol Accumulation in SMCs by Regulating Cholesterol Efflux Through ABCA1

**Figure 4:**
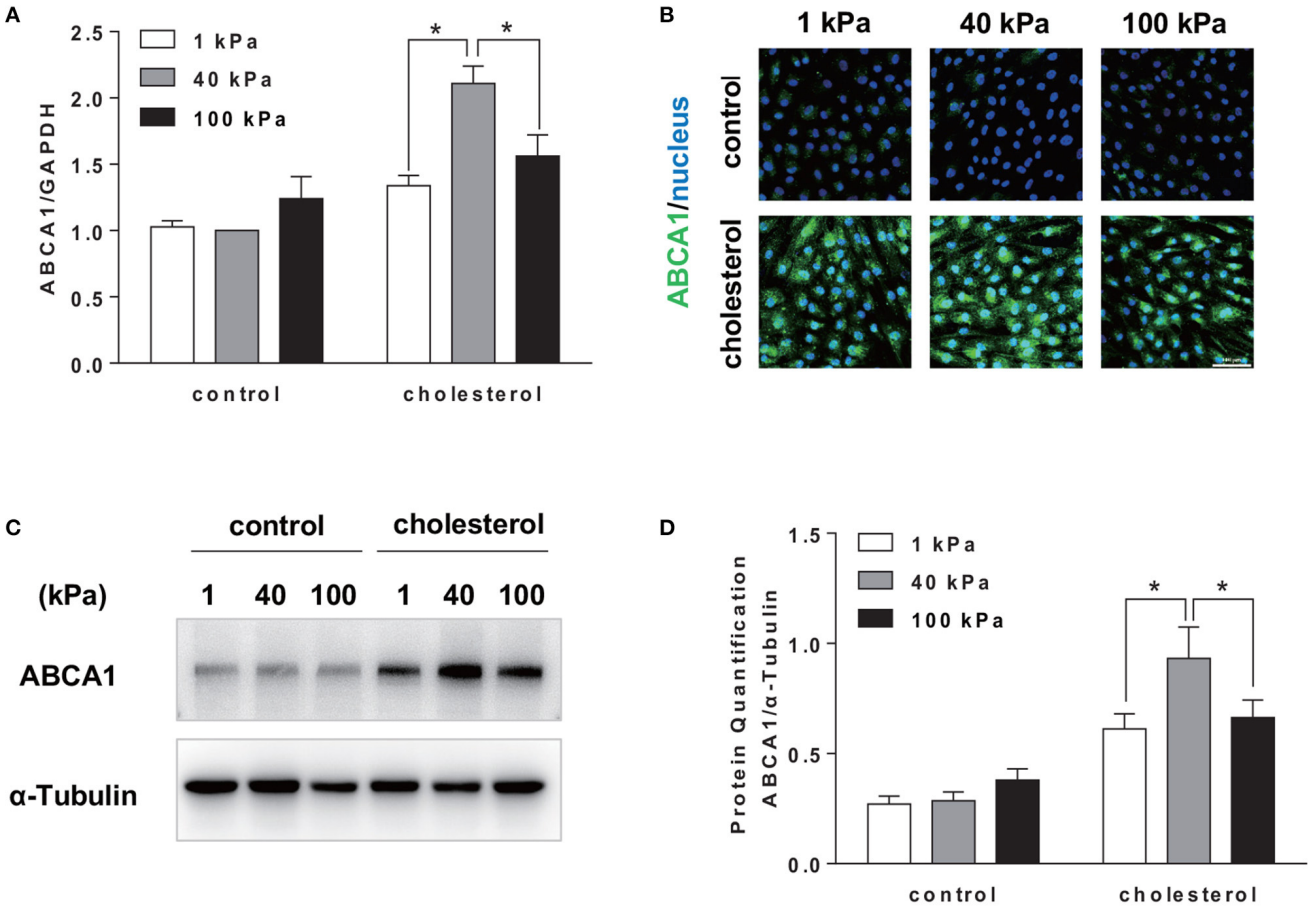
Substrate stiffness affected cholesterol accumulation in SMCs by regulating cholesterol efflux through ABCA1. SMCs were seeded on substrate with different stiffness and treated with 20 μg/ml Chol: MβCD for 72 h. SMCs without treatment were used as control. **(A)** ABCA1 gene expression level was measured by RT-qPCR. GAPDH was used as the internal control for normalization (*n* = 3). **(B)** ABCA1 (green) expression in SMCs cultured on substrate with different stiffness without (control) or with cholesterol treatment (blue: DAPI staining of the nucleus, scale bar = 100 μm). **(C,D)** ABCA1 protein expression level was analyzed by Western blotting analysis. The expression of α-Tubulin was used as the internal control. Quantitation of the protein expression was calculated as the ratio of target protein to α-Tubulin (*n* = 3). ^*^p < 0.05.

Although cellular cholesterol homeostasis is a combined outcome of the uptake, synthesis, and efflux of cholesterol, cholesterol efflux is the key event of RCT to remove excess cholesterol. Cholesterol uptake by SMCs is largely mediated by non-specific pinocytosis (Rivera et al., 2013); therefore, we focused on the effect of substrate stiffness on the regulation of cholesterol efflux. Cholesterol efflux is mediated by ABCA1, an active transmembrane transporter related to the transmembrane transport of cholesterol and phospholipids. Interestingly, we found that ABCA1 mRNA expression on 40 kPa substrate was significantly higher than 1 and 100 kPa substrates (Figure 4A). Immunostaining showed that the expression of ABCA1 is higher on 40 kPa substrate compared to that on 1 and 100 kPa ones (Figure 4B). Western blotting analysis confirmed the protein level of ABCA1 being consistent with the gene expression level (Figures 4C,D). Therefore, the regulatory effect of substrate stiffness on cholesterol accumulation in SMC is related to ABCA1 expression and cholesterol efflux.

### 3.5. LXR Signaling Pathway Converged With Stiffness to Regulate ABCA1 and Cholesterol Efflux in SMCs

**Figure 5:**
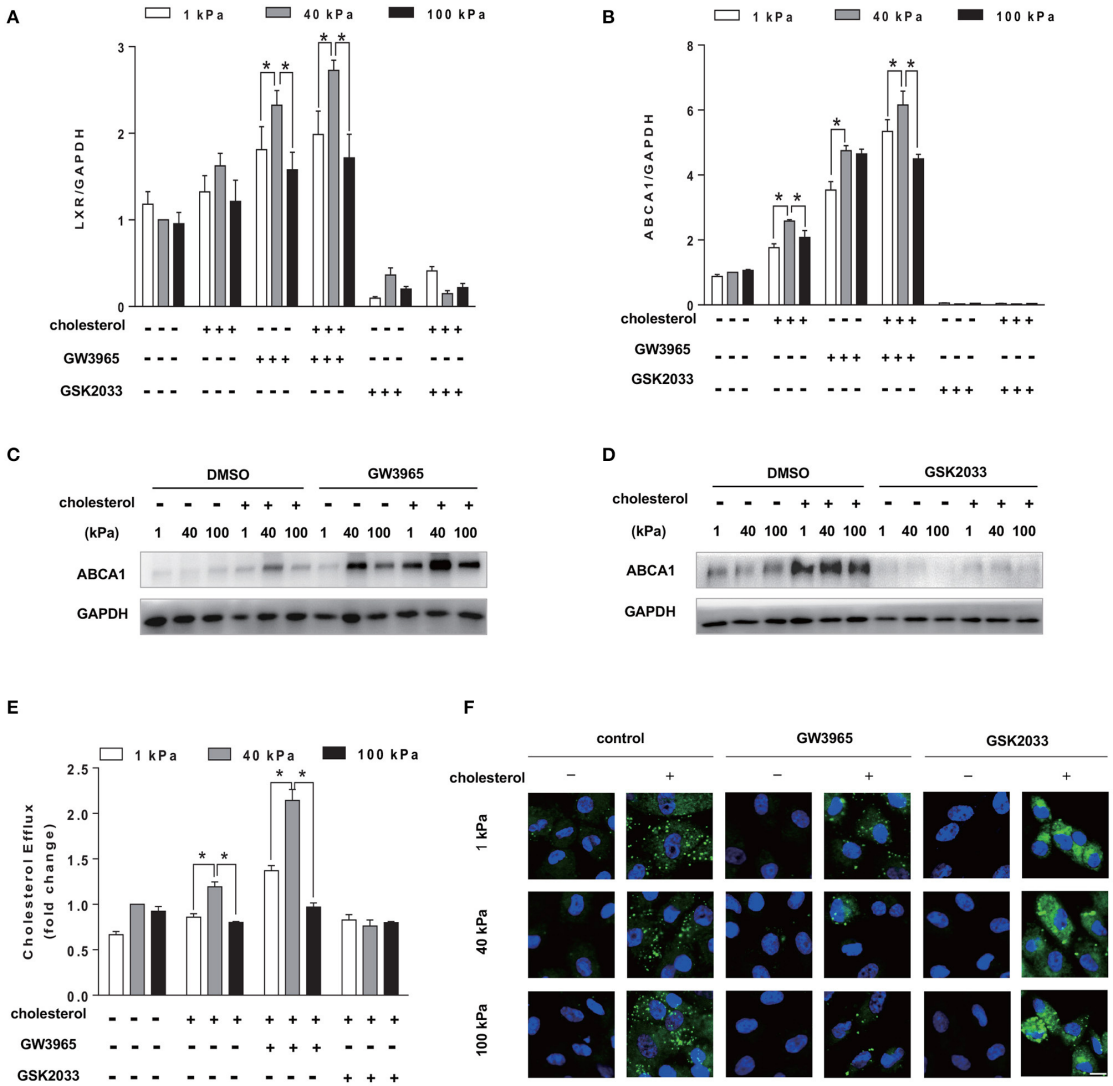
Additive effect of substrate stiffness and LXR signaling pathway on ABCA1 expression and cholesterol efflux. SMCs were seeded on substrate with different stiffness and treated with 20 μg/ml Chol: MβCD and GW3965 (1 μM) or GSK2033 (1 μM) in the serum-free medium for 72 h. **(A,B)** LXR and ABCA1 gene expression level was measured by RT-qPCR. GAPDH was used as the internal control for normalization (*n* = 3). **(C,D)** ABCA1 protein expression was analyzed by Western blotting analysis after GW3965 or GSK2033 treatment. DMSO was used as control. The expression of GAPDH was used as the internal control (*n* = 3). **(E)** Cholesterol efflux rate. The results were expressed as fold change of all groups compared with the control group at 40 kPa (*n* = 3). **(F)** The cholesterol accumulation in SMCs after GW3965 or GSK2033 treatment (green: BODIPY 493/503 staining of cholesterol; blue: DAPI staining of nucleus, scale bar = 10 μm). ^*^p < 0.05.

To further understand the influence of substrate stiffness on SMCs cholesterol efflux, we modulated the LXR signaling pathway, the upstream regulator of ABCA1, to regulate the expression of ABCA1. LXR has been reported as a sterol-responsive nuclear receptor. GW3965 is a selective agonist to LXR and GSK2033 is a selective LXR antagonist (Collins et al., 2002; Delvecchio et al., 2007; Helder et al., 2020). The activation effect of GW3965 and the inhibition effect of GSK2033 on LXR expression was confirmed (Figure 5A). Activation of LXR by GW3965 treatment increased both the mRNA and protein expression of ABCA1 in SMCs (Figures 5B,C). Moreover, the expression level of LXR and ABCA1 on 40 kPa was higher than that on 1 and 100 kPa substrates. In contrast, inhibition of LXR by GSK2033 treatment almost abolished both the mRNA level and protein expression level of ABCA1 on all substrates with different stiffnesses (Figures 5B,D). As a result, with the upregulation of LXR and cholesterol treatment for 72 hours, SMC cholesterol efflux increased significantly (*p* < 0.05). In addition, compared with 1 and 100 kPa, 40 kPa stiffness induced the highest cholesterol efflux rate in SMCs, suggesting the additive effect of substrate stiffness and LXR signaling. In contrast, downregulating LXR by GSK2033 treatment blocked cholesterol efflux of SMCs, regardless of substrate stiffness (Figure 5E). Furthermore, change in cholesterol efflux resulted in the change of the cellular cholesterol content. BODIPY 493/503 staining demonstrated that LXR agonist GW3965 treatment led to very low cholesterol accumulation in SMCs, and there is no observable difference among the different substrate stiffness. However, inhibition of LXR resulted in the inhibition of cholesterol efflux. Consequently, the cellular cholesterol level was increased on all substrate stiffness (Figure 5F).

### 3.6. Knockdown of ABCA1 Increased Cholesterol Accumulation in SMCs

**Figure 6:**
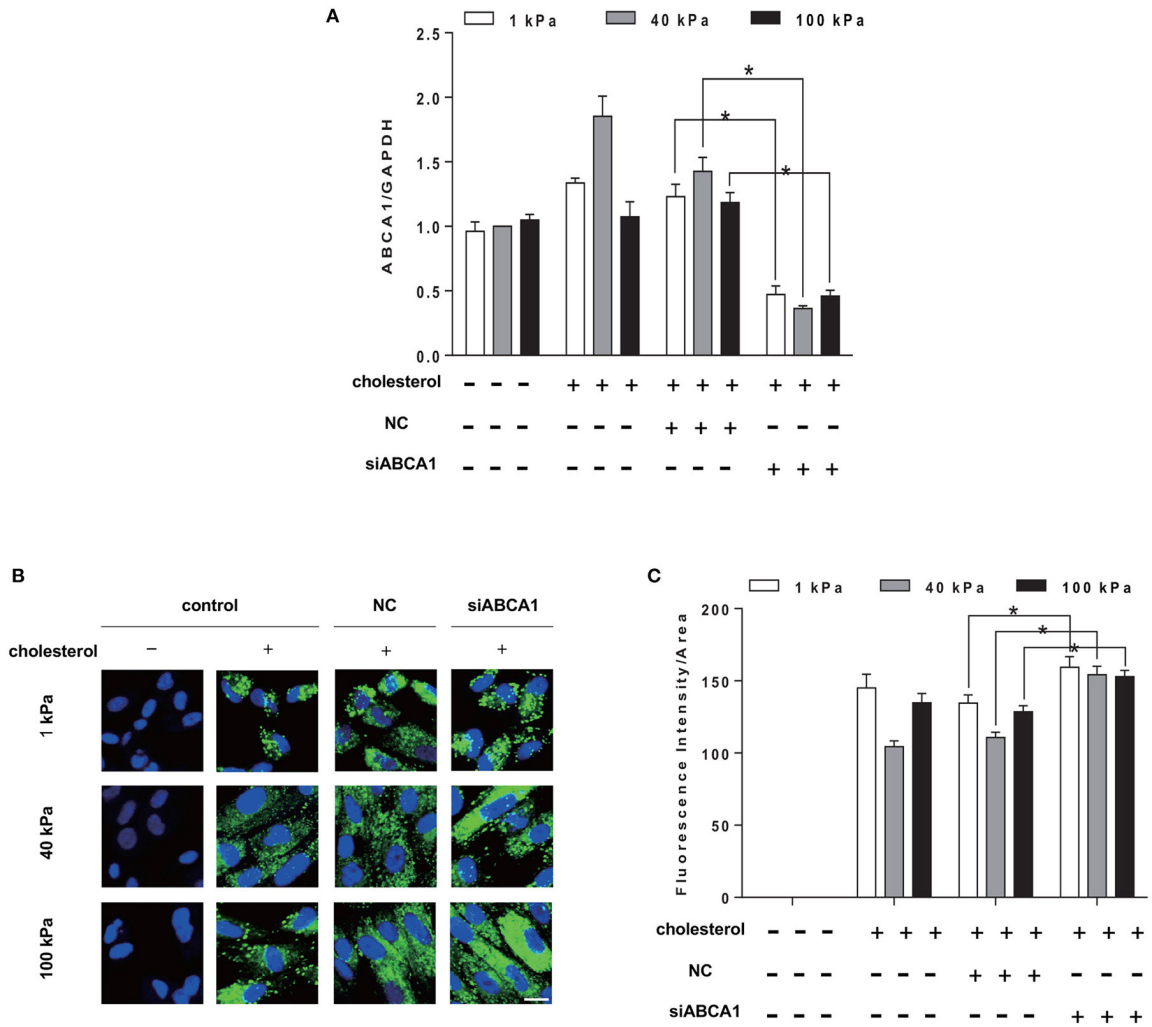
Knockdown of ABCA1 promotes cholesterol accumulation in SMCs. SMCs were seeded on substrate with different stiffness and treated with 20 μg/ml Chol: MβCD and siABCA1 for 72 h. **(A)** ABCA1 gene expression level was measured by RT-qPCR. GAPDH was used as the internal control for normalization (*n* = 3) (NC, Negative siRNA control). **(B)** The cholesterol in SMCs after ABCA1 gene knockdown (green: BODIPY 493/503 staining of cholesterol; blue: DAPI staining of nucleus, scale bar = 10 μm). **(C)** The level of cholesterol quantified by the fluorescence intensity and normalized by the area of the green fluorescence. ^*^p < 0.05.

To further verify that ABCA1 is a key mediator of substrate stiffness regulating cholesterol accumulation in SMCs, we knocked down cholesterol efflux gene ABCA1. The results showed that siABCA1 effectively reduced ABCA1 expression (Figure 6A). BODIPY 493/503 staining results showed that, compared with the control group, the cholesterol accumulation level in SMCs of each substrate stiffness was higher in the siABCA1 group (Figures 6B,C).

## 4. Discussion and Conclusion

SMCs are the main cell component to maintain the vascular wall structure and vascular tension. And their phenotypic transformation is essential to response to environment cues, including cytokines, mechanical influences, and extracellular cholesterol (Owens et al., 2004). In atherosclerosis, SMCs provide an initial location for the retention of cholesterol that facilitates the formation of thickened intimal layer. During this process, complex structural and functional changes occur in the SMCs, resulting in the development of the foam cells enclosing excess cholesterol (Rong et al., 2003; Feil et al., 2014). It has been reported that more than 50% of CD68^+^ foam cells are SMCs-derived in atherosclerotic lesion (Allahverdian et al., 2014). In addition, studies have shown that cholesterol load can induce the expression of macrophage marker CD68 in both mouse and human SMCs, meanwhile reducing the expression of the contractile marker ACTA2 (Rong et al., 2003; Vengrenyuk et al., 2015; Liu et al., 2017; Vendrov et al., 2019). Our results also showed that, cholesterol treatment reduced the expression of ACTA2 in SMCs although the gene and protein expression of ACTA2 still increased with substrate stiffness. On the other hand, the expression level of CD68 was lower at 40 kPa compared to the cells on and 100 kPa, suggesting a different mechanism of stiffness regulation. Taken together, these results suggested that substrate stiffness could regulate SMC phenotypic switching to foam cells and that the physiological level of substrate stiffness could suppress phenotypic changes.

Atherosclerosis is a chronic pathological process, in which the deposition of lipoproteins in the blood vessels leads to the softening of blood vessels and the formation of fat stripes. Subsequently, hyperplasia of fibrous tissue and calcareous deposition result in thickening and hardening of the arterial wall (Lemarié et al., 2010). Studies have shown that the stiffness of early lesion aortic was 10 times less than the normal aortic (Tian et al., 2019). In advanced atherosclerosis, vascular stiffness may increase to 100 kPa or more due to the calcification of arterial wall (Matsumoto et al., 2002). Our results showed that the substrate stiffness directly affected the cholesterol accumulation in SMCs. Among the stiffness range we tested (1, 8, 20, 40, 100 kPa), the cholesterol accumulation in SMCs on both softer (1 and 8 kPa) and harder substrates (100 kPa) demonstrated a significant trend of increase compared to those on substrates with moderate stiffness (20 and 40 kPa). Therefore, in the subsequent research, 1, 40, and 100 kPa substrate stiffness were selected as the experimental conditions. Interestingly, the result from cellular TC analysis confirmed this observation, that TC was also higher on 1 and 100 kPa substrates. This is a direct evidence that blood vessel stiffness within physiological to pathological range significantly affect SMCs cholesterol content.

Since cholesterol accumulation in the arterial wall is an important characteristic of atherosclerosis, RCT is considered to play a key role of anti-atherosclerosis. RCT can remove excess cholesterol from peripheral tissues to the liver (Liao and McLachlan, 2018). Specifically, apolipoprotein A1 (ApoA1) obtains free cholesterol through ABCA1 from peripheral cells and free cholesterol can be esterified into cholesterol ester by lecithin-cholesterol acyl transferase (LCAT) to mature high density lipoprotein (HDL). HDL can be selectively absorbed by liver. Finally, liver excretes cholesterol into feces or converted it into bile acid (Tall et al., 2008; Talbot et al., 2018). Studies have shown that the loss of ABCA1 and ABCG1 resulted in a significant decrease in the cholesterol efflux from macrophages (Yvan-Charvet et al., 2007). Consistently, our results demonstrated that the change in the expression level of ABCA1 resulted in the change in cholesterol content in the SMCs. Although ABCG1 is also responsible for cholesterol efflux, our results showed that there were only slight changes in the expression of ABCG1 among substrate stiffness compared to that of ABCA1 (Supplementary Figure 1). Moreover, substrate stiffness was directly responsible for the ABCA1 expression level change. Therefore, activation of RCT by enhancing ABCA1 expression through manipulating substrate stiffness may be an attractive strategy to alleviate cholesterol accumulation in the SMCs and prevent atherosclerosis.

LXR is the key regulator of lipid balance in mammals and plays an important role in regulating cholesterol transportation and lipid metabolism. Upon activation, LXR can induce the expression of membrane transporters, thereby promoting cholesterol efflux and preventing cholesterol overload (Choi et al., 2009; Hong and Tontonoz, 2014). It is reported that activation of LXR could induce the expression of ABCA1 in primary human airway SMCs, increasing cholesterol efflux. In addition, the RCT rate of macrophages increased after the treatment with synthetic LXR-RXR ligands T1317 (Delvecchio et al., 2007), which is in agreement with our results. Our results show that LXR agonist, GW3965, upregulated the expression level of ABCA1 and subsequently cholesterol efflux showing an additive effect with 40 kPa substrate stiffness. On the other hand, blocking cholesterol efflux by either using LXR antagonist GSK2033 or knocking down ABCA1 led to cholesterol retention inside SMCs. Although ABCA1 expression was higher at 40 kPa substrate stiffness, blocking ABCA1 expression by LXR abolished the stiffness effect, suggesting that ABCA1 is an important mediator of stiffness effect on cholesterol efflux.

In conclusion, our study demonstrates that different substrate stiffness regulates SMC phenotype switching in response to high extracellular cholesterol level. Both 1 and 100 kPa substrate stiffness increases cholesterol retention in SMCs by suppressing the cholesterol efflux, while intermediate substrate stiffness at 40 kPa results in the least cholesterol accumulation and helps maintain a contractile phenotype. Moreover, our results suggest that cholesterol efflux gene ABCA1 is a key mediator of substrate stiffness regulation of cholesterol accumulation in SMCs. These findings provide further insight into the biophysical regulation of cholesterol metabolism and SMC phenotype. In future studies, it would be interesting to explore how substrate stiffness transmits mechanical signals into SMCs and affects cholesterol accumulation or efflux.

## Data Availability Statement

The datasets presented in this study can be found in online repositories. The names of the repository/repositories and accession number(s) can be found at: https://www.ncbi.nlm.nih.gov/, NM 001256799.3; https://www.ncbi.nlm.nih.gov/, NM 005502; https://www.ncbi.nlm.nih.gov/, NM 001141945.2; https://www.ncbi.nlm.nih.gov/, NM 001040059; https://www.ncbi.nlm.nih.gov/, NM 001357678.

## Author Contributions

All authors have made significant contributions to the work and analysis and interpretation of data. SL, YZ, and XM conception or design of the work and manuscript writing. XM, YT, and HW acquisition of the data. All authors have approved the submitted version and have agreed both to be personally accountable for the author's own contributions and to ensure that questions related to the accuracy or integrity of any part of the work.

## Conflict of Interest

The authors declare that the research was conducted in the absence of any commercial or financial relationships that could be construed as a potential conflict of interest.
