# Supplementary material for: Substrate Stiffness Regulates Cholesterol Efflux in Smooth Muscle Cells
Source: Front Cell Dev Biol. 2021 May 18;9:648715. doi: 10.3389/fcell.2021.648715 (PMC8168435; doi:10.3389/fcell.2021.648715)
Supplement: Supplementary file 1 [file Image_1.pdf]

Supplementary Material

1 SUPPLEMENTARY FIGURE

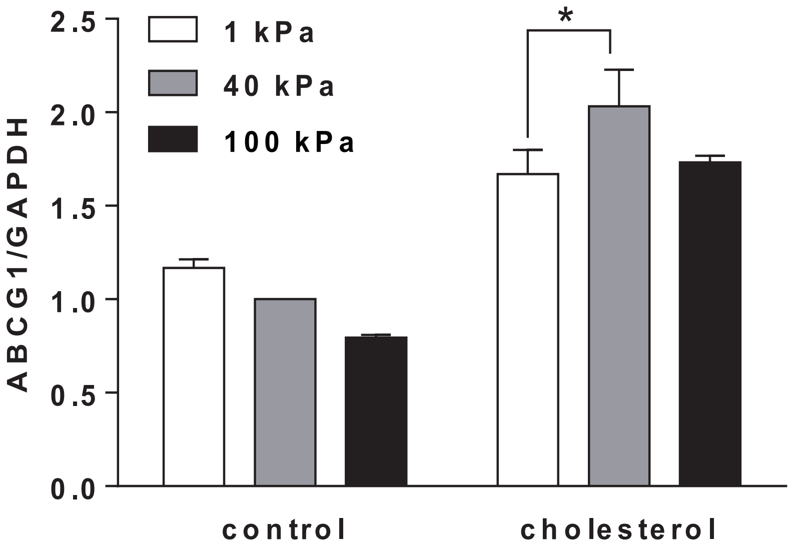

Figure S1. The expression of cholesterol efflux gene ABCG1 in SMCs. ABCG1 gene expression level was measured by RT-qPCR. GAPDH was used as the internal control for normalization (n = 3). * indicates $p < 0.05$.
